# Liver-related long-term outcomes of alpha-glucosidase inhibitors in patients with diabetes and liver cirrhosis

**DOI:** 10.3389/fphar.2022.1049094

**Published:** 2022-12-21

**Authors:** Fu-Shun Yen, Ming-Chih Hou, James Cheng-Chung Wei, Ying-Hsiu Shih, Chung Y. Hsu, Chih-Cheng Hsu, Chii-Min Hwu

**Affiliations:** ^1^ Yen’s Clinic, Taoyuan, Taiwan; ^2^ Division of Gastroenterology and Hepatology, Department of Medicine, Taipei Veterans General Hospital, Taipei, Taiwan; ^3^ Institute of Clinical Medicine, School of Medicine, National Yang-Ming Chiao Tung University, Taipei, Taiwan; ^4^ Department of Allergy, Immunology and Rheumatology, Chung Shan Medical University Hospital, Taichung, Taiwan; ^5^ Institute of Medicine, Chung Shan Medical University, Taichung, Taiwan; ^6^ Graduate Institute of Integrated Medicine, China Medical University, Taichung, Taiwan; ^7^ Management Office for Health Data, China Medical University Hospital, Taichung, Taiwan; ^8^ College of Medicine, China Medical University, Taichung, Taiwan; ^9^ Graduate Institute of Biomedical Sciences, China Medical University, Taichung, Taiwan; ^10^ Institute of Population Health Sciences, National Health Research Institutes, Taipei, Miaoli, Taiwan; ^11^ Department of Health Services Administration, China Medical University, Taichung, Taiwan; ^12^ Department of Family Medicine, Min-Sheng General Hospital, Taoyuan, Taiwan; ^13^ National Center for Geriatrics and Welfare Research, National Health Research Institutes, Taipei, Miaoli, Taiwan; ^14^ Section of Endocrinology and Metabolism, Department of Medicine, Taipei Veterans General Hospital, Taipei, Taiwan

**Keywords:** all-cause mortality, decompensated cirrhosis, hepatic encephalopathy, hepatic failure, hepatocellular carcinoma

## Abstract

**Background:** Adequate management of diabetes in patients with liver cirrhosis can be challenging. We conducted this study to investigate the liver-related long term outcomes of alpha-glucosidase inhibitors (AGIs) in patients with diabetes and cirrhosis.

**Methods:** From National Health Insurance Research Database (NHIRD) in Taiwan, we recruited propensity-score matched alpha-glucosidase inhibitor users and non-users from a cohort of type 2 diabetes mellitus (T2DM) with compensated liver cirrhosis between 1 January 2000, and 31 December 2017, and followed them until 31 December 2018. Cox proportional hazards models with robust sandwich standard error estimates were used to assess the risk of main outcomes for alpha-glucosidase inhibitor users versus non-users.

**Results:** The incidence rates of mortality during follow-up were 65.56 vs. 96.06 per 1,000 patient-years for alpha-glucosidase inhibitor users and non-users, respectively. The multivariable-adjusted model shows that alpha-glucosidase inhibitor users had significantly lower risks of all-cause mortality (aHR 0.63, 95% CI 0.56–0.71), hepatocellular carcinoma (aHR 0.55, 95% CI 0.46–0.67), decompensated cirrhosis (aHR 0.74 95% CI 0.63–0.87), hepatic encephalopathy (aHR 0.72, 95% CI 0.60–0.87), and hepatic failure (aHR 0.74, 95% CI 0.62–0.88) than alpha-glucosidase inhibitor non-users. Patients who received alpha-glucosidase inhibitors for a cumulative duration of more than 364 days had significantly lower risks of these outcomes than non-users.

**Conclusion:** Alpha-glucosidase inhibitor use was associated with a lower risk of mortality, hepatocellular carcinoma, decompensated cirrhosis, and hepatic failure in patients with diabetes and compensated cirrhosis. alpha-glucosidase inhibitors may be useful for the management of diabetes in patients with compensated liver cirrhosis. Large-scale prospective studies are required to verify our results.

## 1 Introduction

Cirrhosis is characterized by repeated injury, necroinflammation, nodular regeneration surrounded by fibrotic septa, parenchyma extinction, and distortion of hepatic vascular architecture (Tsochatzis et al., 2014). It is the late stage of chronic liver disease. Hepatitis B or C virus infection, alcohol drinking, and non-alcoholic fatty liver disease can alone or synergistically engender liver cirrhosis (Tsochatzis et al., 2014). Aproximately 1,690 million people worldwide have cirrhosis, and about 1.47 million people died of cirrhosis in 2019 (GBD, 2019). Due to the past hepatitis B virus (HBV) epidemic, cirrhosis is not uncommon in Taiwan, with approximately 7.84 million patients with cirrhosis (nearly 23.7% of these resulting from HBV infection) (GBD 2019).

Liver cirrhosis can reduce insulin extraction, coupled with portal-systemic shunts, and leads to systemic hyperinsulinemia and insulin resistance (Elkrief et al., 2016; Garcia-Compean et al., 2009). Moreover, the metabolic homeostasis of glucose is impaired in liver cirrhosis (Elkrief et al., 2016; Petrides et al., 1994). Therefore, approximately 60%–80% of patients with cirrhosis have glucose intolerance, and 30% have diabetes mellitus (Elkrief et al., 2016; Petrides et al., 1994). Diabetes can worsen the clinical course and mortality risk of liver cirrhosis (Elkrief et al., 2016). Therefore, adequate glucose control in patients with liver cirrhosis is imperative. However, it is complicated to adequately manage diabetes and choose antidiabetic drugs due to suboptimal nutritional status and altered drug metabolism (Ahmadieh and Azar, 2014; Garcia-Compean et al., 2009) in patients with liver cirrhosis (Gangopadhyay and Singh, 2017; Tolman et al., 2007).

Alpha-glucosidase inhibitors (AGIs) exhibit reversible and competitive inhibition of alpha-glucosidases in the brush border of small intestines, delay complex carbohydrate digestion in upper small intestine, retard glucose absorption, and lower postprandial glycemia (AGIs can decrease hemoglobin A1C by approximately 0.8% and postprandial glucose approximately 41.4 mg/dl) (Standl and Schnell, 2012). As cirrhosis is mainly associated with postprandial hyperglycemia (Garcia-Compean et al., 2009), AGIs may be suitable for treating diabetes in patients with cirrhosis (Gangopadhyay and Singh, 2017) Moreover, AGIs do not undergo hepatic metabolism nor modify liver function in patients with cirrhosis (Gentile et al., 2001). Studies have demonstrated that AGIs are safe and effective in alcoholic cirrhosis (Zillikens et al., 1989), non-alcoholic cirrhosis (Gentile et al., 2005), and patients with cirrhosis receiving insulin injections (Gentile et al., 2001). One randomized trial showed that acarbose decreased blood ammonia levels and improved cognitive function in patients with cirrhosis and mild hepatic encephalopathy (Gentile et al., 2005). However, these are all small-scale, short-term studies. Therefore, we conducted this nationwide cohort study to investigate the liver-related long-term outcomes of AGIs in patients with diabetes and liver cirrhosis.

## 2 Materials and methods

### 2.1 Data source

Taiwan implemented the National Health Insurance (NHI) program in 1995. Under this program, the government and employers pay most of the premium, while the public pays a small amount. The government is the single buyer. By 2000, approximately 95% of 23 million people in Taiwan were covered by the NHI program (Cheng, 2003). The NHI Research Database (NHIRD) records residence, sex, age, payroll, prescription, medical procedures, and disease diagnosis according to the International Classification of Diseases [Ninth and 10th Revision, Clinical Modification (ICD-9-CM and ICD-10-CM)]. The NHIRD is linked with the National Death Registry to certify death information. All methods applied in the present study were conducted in accordance with the Declaration of Helsinki. This study was approved by the Research Ethics Committee of China Medical University and Hospital [CMUH110-REC1-038(CR-1)]. All identifiable information of caregivers and participants was scrambled and encrypted before release, and we have had permission by the Research Ethics Committee to waive the informed consent of the participants.

### 2.2 Study design

**FIGURE 1:**
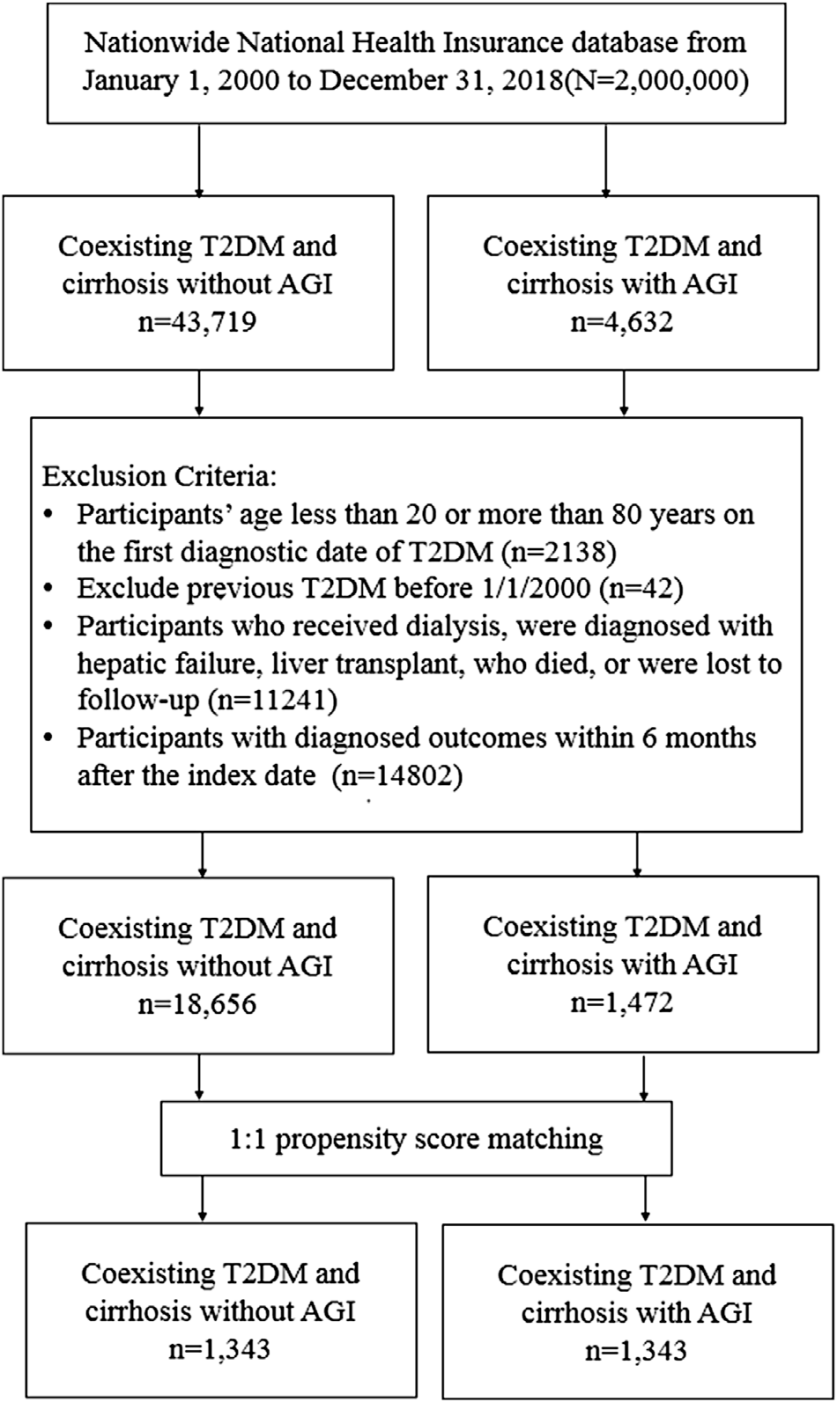
Flowchart of the selected study participants.

We recruited participants from the NHIRD with type 2 diabetes mellitus (T2DM) and liver cirrhosis between 1 January 2000, and 31 December 2017, and followed them until 31 December 2018 (Figure 1). T2DM was defined by the ICD-9-CM code 250. xx or ICD-10-CM: E11; liver cirrhosis was ascertained using the ICD codes (Supplementary Table S1) for at least two outpatient diagnoses in 1 year or one hospitalization. The algorithm of using ICD codings to define T2DM and liver cirrhosis has been validated by previous studies (Lin et al., 2005; Nehra et al., 2013), with a diagnostic accuracy of 74.6% and 82.6% for T2DM and cirrhosis, respectively. Decompensated cirrhosis was defined in participants with liver cirrhosis and variceal bleeding, ascites, hepatic encephalopathy, or jaundice (Mukerji et al., 2012), while compensated cirrhosis was defined in participants without these complications. We excluded participants younger than 20 or older than 80 years, missing sex, diagnoses of type 1 diabetes mellitus, hepatocellular carcinoma (HCC), decompensated cirrhosis, hepatic failure, liver transplant, or dialysis. We also excluded participants who died, were lost to follow-up, received a diagnosis of HCC, esophageal varices with bleeding, ascites, hepatic encephalopathy, jaundice, or hepatic failure within 6 months after the index date to avoid latent morbidity or mortality. We excluded participants with T2DM diagnosed in 1997–1999 to exclude prevalent diseases.

### 2.3 Procedures

**FIGURE 2:**
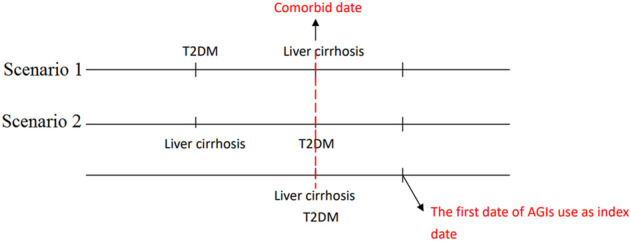
The scenarios for the concurrent diagnosis of T2DM and liver cirrhosis and the index date.

We defined the date of concurrent diagnosis of T2DM and liver cirrhosis as the comorbid date (Figure 2). Patients receiving AGIs after the comorbid date were defined as AGI users, and those who never received AGIs during the study period were defined as non-users. The first date of AGI use after the comorbid date was defined as the index date, and we recorded the index date of the control cases with the same period of T2DM diagnosis for AGI use. We identified potentially confounding variables, including age, sex, smoking status, comorbidities diagnosed within 1 year before the index date, and medications [e.g., antidiabetic medications, antihypertensive drugs, statin, and aspirin. Glucagon-like peptide one receptor agonist (GLP-1 RA) was launched in Taiwan since 2011, but the number of patients with type 2 diabetes using this drug is relatively small, especially in patients with liver cirrhosis, so we did not consider GLP-1 RA in this study]. The comorbid disorders identified in this study include alcohol-related disorders, hepatitis B virus (HBV) infection, hepatitis C virus (HCV) infection, hypertension, dyslipidemia, chronic kidney disease (CKD), and chronic obstructive pulmonary disease (COPD). To truly reflect the characteristics of participants with T2DM and liver cirrhosis, we used the Charlson Comorbidity Index (CCI) to quantify participant comorbidity profiles (Meduru et al., 2007) and the Diabetes Complications Severity Index (DCSI) score (Young et al., 2008; Wicke et al., 2019) for diabetes severity.

### 2.4 Main outcomes

The main outcomes of this study include 1) all-cause mortality, identified from the National Death Registry link; 2) HCC (by ICD codings); 3) decompensated cirrhosis (a composite including variceal bleeding, ascites, jaundice, or hepatic encephalopathy (Mukerji et al., 2012); 4) esophageal varices with bleeding; 5) ascites; 6) hepatic encephalopathy; 7) hepatic failure characterized by coagulopathy, hepatic encephalopathy, with or without multiorgan failure (Bernal and Wendon, 2013). To calculate the risk of mortality and other relevant outcomes, we censored participants on the first occurrence of death, respective outcomes, or at the end of follow-up on 31 December 2018.

### 2.5 Statistical analysis

We adopted propensity score matching to optimize the comparability between AGIs users and non-users (D’Agostino, 1998). The propensity score was calculated for every participant by the non-parsimonious multivariable logistic regression, with the receipt of AGIs as the dependent variable. We included 25 potentially related covariates in the analysis as independent variables (Table 1). The nearest-neighbor algorithm was used to construct matched pairs with the assumption that a *p*-value of >.05 indicates a negligible difference between the study and comparison groups (Iezzoni, 1997).

**TABLE 1:** Comparison of baseline characteristics in persons with coexisting T2DM and cirrhosis with and without AGI use.

Variables	Coexisting T2DM and cirrhosis without AGI		Coexisting T2DM and cirrhosis with AGI		*p*-value
	(*N* = 1,343)		(*N* = 1,343)		
	n	%	n	%	
Sex					
female	434	32.32	424	31.57	0.68
male	909	67.68	919	68.43	0.68
Age					
18–40	89	6.63	100	7.45	0.48
40–60	730	54.36	745	55.47	0.48
60–80	524	39.02	498	37.08	0.48
mean, (SD)^a^	56.93	11.06	56.43	10.97	0.24
Smoking	39	2.90	36	2.68	0.73
Comorbidities					
Alcohol-related disorders	327	24.35	317	23.60	0.65
Hepatitis B	243	18.09	220	16.38	0.24
Hepatitis C	200	14.89	194	14.45	0.74
Hypertension	817	60.83	762	56.74	0.03
Dyslipidemia	688	51.23	669	49.81	0.46
Chronic kidney disease	230	17.13	223	16.60	0.72
COPD	220	16.38	211	15.71	0.64
CCI					
1	835	62.17	848	63.14	0.75
2–3	319	23.75	319	23.75	0.75
>3	189	14.07	176	13.11	0.75
Medications					
Metformin	1,060	78.93	1,060	78.93	1.00
Sulfonylureas	1,038	77.29	1,034	76.99	0.85
Meglitinides	30	2.23	36	2.68	0.45
TZD	204	15.19	213	15.86	0.63
DPP-4 inhibitors	151	11.24	166	12.36	0.37
SGLT2 inhibitors	7	0.52	7	0.52	1.00
Insulin	711	52.94	670	49.89	0.11
ACEI/ARB	672	50.04	655	48.77	0.51
β-blockers	448	33.36	438	32.61	0.68
Calcium-channel blockers	686	51.08	657	48.92	0.26
Diuretics	647	48.18	607	45.20	0.12
Statin	381	28.37	375	27.92	0.80
Aspirin	460	34.25	452	33.66	0.74
DCSI					
0	580	43.19	572	42.59	0.49
1	305	22.71	286	21.30	0.49
≥2	458	34.10	485	36.11	0.49

COPD, chronic obstructive pulmonary disease; CCI, charlson comorbidity index; DCSI, diabetes complications severity index; TZD, thiazolidinedione; DPP-4, dipeptidyl peptidase-4; SGLT2, sodium-glucose cotransporter 2; ACEI, angiotensin-converting enzyme inhibitor; ARB, angiotensin receptor blocker. Data shown as n (%) or mean ± SD.

^a^ : Student’s *t*-test. A *p*-value >0.05 indicates a negligible difference.

Crude and multivariable-adjusted Cox proportional hazards models with robust sandwich standard error estimates were used to compare the outcomes between AGIs users and non-users. All analyses were performed on an intention-to-treat basis. The results were represented as hazard ratios (HR) and 95% confidence interval (CI) for AGIs users compared with non-users. We checked the proportional-hazards assumption using tests based on the Schoenfeld residuals and complementary log-log plots. We compared the cumulative incidences of mortality, decompensated cirrhosis, and hepatic failure over time between AGIs users and non-users using the Kaplan-Meier method and the log-rank test.

To investigate the dose-response relationship, we analyzed the risks of mortality, HCC, decompensated cirrhosis, hepatic encephalopathy, and hepatic failure by three cumulative durations of AGI use (<182, 182–364, >364 days) relative to nonuse of AGIs.

A two-tailed *p*-value less than .05 was considered statistically significant. SAS version 9.4 and Stata SE version 11.0 were used for analysis.

## 3 Results

From 1 January 2000, to 31 December 2017, a total of 48,351 patients were diagnosed with T2DM and compensated liver cirrhosis; 4,632 patients were AGI users, and 43,719 patients were AGI non-users. The flowchart of the present study is shown in Figure 1.

After propensity score matching, 1,343 diabetes patients with compensated liver cirrhosis were selected. The matched pairs were approximate for all measured variables. The mean age of the cohort was 56.68 years; the mean proportion of females was 31.95%; the prevalence of HBV and HCV infections was 17.24% and 14.67%, respectively, and the mean follow-up time for AGI users and non-users was 6.74 and 5.18 years, respectively.

In the matched cohort of T2DM with compensated liver cirrhosis, 609 AGIs users and 684 non-users died during follow-up (incidence rates of 65.56 vs. 96.06 per 1,000 patient-years). The multivariable-adjusted model shows that AGI users exhibited significantly lower mortality risk (aHR 0.63, 95% CI 0.56–0.71; Table 2).

**TABLE 2:** Hazard ratios and confidence intervals for the outcomes between AGI users and non-users.

Outcome	Non-AGI						AGI		*p*-value	aHR^a^	(95% CI)	*p*-value
	n	PY	IR	n	PY	IR	cHR	(95% CI)				
Death	684	7,121	96.06	609	9,289	65.56	0.66	(0.59, 0.74)	<0.001	0.63	(0.56, 0.71)	<0.001
Hepatocellular carcinoma	270	6,716	40.20	200	8,888	22.50	0.55	(0.46, 0.66)	<0.001	0.55	(0.46, 0.67)	<0.001
Decompensated cirrhosis	313	6,754	46.34	306	8,675	35.27	0.77	(0.66, 0.9)	0.001	0.74	(0.63, 0.87)	<0.001
Variceal bleeding	19	7,074	2.69	28	9,183	3.05	1.15	(0.64, 2.06)	0.64	1.16	(0.64, 2.1)	0.63
Ascites	105	6,999	15.00	118	9,071	13.01	0.86	(0.66, 1.12)	0.25	0.82	(0.63, 1.08)	0.15
Hepatic encephalopathy	226	6,954	32.50	219	8,956	24.45	0.76	(0.63, 0.91)	0.003	0.72	(0.6, 0.87)	<0.001
Hepatic failure	263	6,907	38.08	262	8,909	29.41	0.77	(0.65, 0.92)	0.003	0.74	(0.62, 0.88)	<0.001

AGI, Alpha-glucosidase inhibitor; PY, person-years; IR, incidence rate, per 1,000 person-years; cHR, crude hazard ratio; aHR, adjusted hazard ratio. aHR^**a**^ multivariable analysis including sex, age, smoking, comorbidities, medications, CCI and DCSI as shown in Table 1.

As shown in Table 2, compared to the non-users, AGI users showed significantly lower risks of HCC (aHR 0.55, 95% CI 0.46–0.67), decompensated cirrhosis (aHR 0.74 95% CI 0.63–0.87), hepatic encephalopathy (aHR 0.72, 95% CI 0.60–0.87), and hepatic failure (aHR 0.74, 95% CI 0.62–0.88) but with no significant difference in the risks of esophageal varices and bleeding (aHR 1.16, 95% CI 0.64–2.1) and ascites (aHR0.82, 95% CI 0.63–1.08). We performed an additional analysis of comparing acarbose and miglitol with non-AGI use on liver-related outcomes (Supplementary Table S2). The vast majority of AGI use in Taiwan is acarbose, but it seems that both acarbose and miglitol have a consistent effect on liver-related outcomes.

**FIGURE 3:**
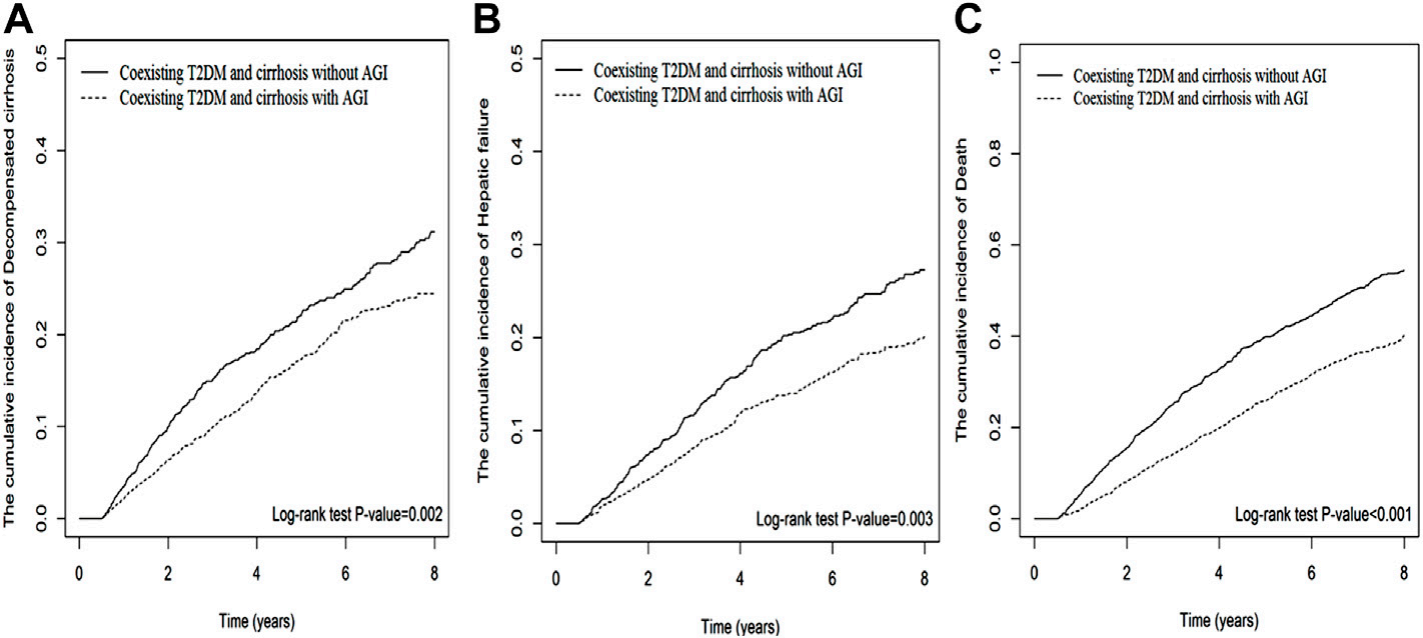
The cumulative incidences of decompensated cirrhosis **(A)**, hepatic failure **(B)**, and death **(C)** between AGI users and non-users in patients with T2DM and compensated liver cirrhosis.

AGI users also showed significantly lower risks in the cumulative incidences of mortality (Log-rank *p* < 0.001), HCC (Log-rank *p* < 0.001), decompensated cirrhosis (Log-rank *p* = 0.002), and hepatic failure (Log-rank *p* = 0.003; Figure 3).

Compared with non-users, those who received AGIs for a cumulative duration of more than 364 days had significantly lower risks of mortality [aHR 0.42 (0.36, 0.5)], HCC [aHR 0.41 (0.31, 0.53)], decompensated cirrhosis [aHR 0.58 (0.46, 0.72)], hepatic encephalopathy [aHR 0.53 (0.41, 0.69)], and hepatic failure [aHR 0.52 (0.41, 0.66)] (Table 3).

**TABLE 3:** The risk of different outcomes with the cumulative duration of AGI use.

Death							
Variables	n	PY	IR	cHR	(95% CI)	aHR^a^	(95% CI)
Nonuse of AGI	684	7,121	96.06	1.00	(References)	1.00	(References)
AGI drug days							
<182	298	3,594	82.91	0.85	(0.74, 0.98)*	0.84	(0.74, 0.97)*
182–364	110	1,362	80.76	0.83	(0.68, 1.01)	0.77	(0.63, 0.95)*
>364	201	4,332	46.40	0.46	(0.39, 0.54)***	0.42	(0.36, 0.5)***

Hepatocellular carcinoma							
Variables	n	PY	IR	cHR	(95% CI)	aHR	(95% CI)
Nonuse of AGI	270	6,716	40.20	1.00	(References)	1.00	(References)
AGI drug days							
<182	82	3,422	23.97	0.59	(0.46, 0.76)***	0.63	(0.49, 0.81)***
182–364	44	1,288	34.16	0.84	(0.61, 1.16)	0.89	(0.64, 1.23)
>364	74	4,178	17.71	0.43	(0.33, 0.56)***	0.41	(0.31, 0.53)***

Decompensated cirrhosis							
Variables	n	PY	IR	cHR	(95% CI)	aHR	(95% CI)
Non-use of AGI	313	6,754	46.34	1.00	(References)	1.00	(References)
AGI drug days							
<182	136	3,342	40.70	0.89	(0.73, 1.09)	0.88	(0.72, 1.07)
182–364	57	1,289	44.22	0.96	(0.73, 1.28)	0.94	(0.7, 1.25)
>364	113	4,044	27.94	0.61	(0.49, 0.76)***	0.58	(0.46, 0.72)***

Hepatic encephalopathy							
Variables	n	PY	IR	cHR	(95% CI)	aHR	(95% CI)
Nonuse of AGI	226	6,954	32.50	1.00	(References)	1.00	(References)
AGI drug days							
<182	100	3,459	28.91	0.89	(0.7, 1.13)	0.88	(0.69, 1.11)
182–364	42	1,325	31.69	0.98	(0.7, 1.36)	0.94	(0.67, 1.31)
>364	77	4,171	18.46	0.57	(0.44, 0.74)***	0.53	(0.41, 0.69)***

Hepatic failure							
Variables	n	PY	IR	cHR	(95% CI)	aHR	(95% CI)
Nonuse of AGI	263	6,907	38.08	1.00	(References)	1.00	(References)
AGI drug days							
<182	121	3,428	35.30	0.93	(0.75, 1.15)	0.92	(0.74, 1.14)
182–364	52	1,312	39.64	1.04	(0.77, 1.4)	1.01	(0.75, 1.36)
>364	89	4,169	21.35	0.56	(0.44, 0.71)***	0.52	(0.41, 0.66)***

AGI, Alpha-glucosidase inhibitor; PY, person-years; IR, incidence rate, per 1,000 person-years; cHR, crude hazard ratio; aHR, adjusted hazard ratio. aHR^a^ multivariable analysis including sex, age, smoking, comorbidities, medications, CCI and DCSI as shown in Table 1. *p < .05, ***p < .001.

## 4 Discussion

This study demonstrated that AGI use was associated with significantly lower risks of decompensated cirrhosis, hepatic encephalopathy, hepatic failure, HCC, and all-cause mortality compared with nonuse of AGIs in patients with compensated liver cirrhosis. Furthermore, a longer duration of AGI use was associated with lower risks of these outcomes.

Gentile et al. (2001) conducted several excellent studies showing that acarbose was safe and effective in patients with non-alcoholic liver cirrhosis (Gentile et al., 2005). The studies also demonstrated that acarbose could reduce blood ammonia levels (52.6%) and improve hepatic encephalopathy (Gentile et al., 2001; Gentile et al., 2005). Our study similarly showed that patients using AGIs exhibited a lower risk of hepatic encephalopathy than AGI non-users [aHR 0.72 (0.6–0.87)]. Our research also showed that AGI users had a lower risk of decompensated cirrhosis [aHR 0.74 (0.63–0.87)], and a longer duration of AGI use was associated with lower risks of hepatic encephalopathy and decompensated cirrhosis. The possible explanations for these results may be 1) AGI use can reduce the proliferation of intestinal proteolytic bacteria and stimulate the proliferation of saccharolytic bacteria, resulting in reduced blood ammonia levels (Gentile et al., 2005). 2) AGIs can delay the metabolism of complex carbohydrates in the small intestine, transfer these undigested carbohydrates to the lower bowel, and increase the peristalsis of the gut; this laxative effect may decrease bacterial overgrowth and diminish ammonia levels in the bowel (Gentile et al., 2005). 3) AGI use can increase intestinal GLP-1 release. GLP-1 may alter nitric oxide production and portal pressure, then affect the risk of cirrhotic decompensation (Seifarth et al., 1998). 4) AGIs may decrease body weight, systolic blood pressure, and portal pressure (Hanefeld et al., 2004; Standl et al., 2014). 5) AGIs may improve postprandial hyperglycemia, decrease oxidative stress, reduce systemic inflammation, lower plasminogen activator inhibitor-1 and fibrinogen levels, and attenuate coagulation activation (Hanefeld et al., 2004; Standl et al., 2014), which may mitigate the risk of cirrhotic decompensation.

There are few case reports of hepatotoxicity treated by AGIs (Gangopadhyay and Singh, 2017; Gentile et al., 2001). However, most clinical studies have shown that AGIs are rarely associated with liver damage (Gentile et al., 1999; Kao et al., 2016). It is conceivable that only a small amount (1%) of acarbose is absorbed and not metabolized by the liver (Gangopadhyay and Singh, 2017; Gentile et al., 2001). Moreover, our study showed that AGIs could decrease the risk of hepatic failure [aHR 0.74 (0.62–0.88)], probably because AGI use is associated with lower risks of hepatic encephalopathy and decompensated cirrhosis, which may then mitigate the risks of hepatic coma and hepatic failure-related complications in this study.

Cirrhosis is responsible for 70%–90% of HCC cases (Blachier et al., 2013). Diabetes may also result in a two to fourfold increase in the risk of liver cancer (Garcia-Compean et al., 2009; Tolman et al., 2007). This study revealed that AGI use was associated with a 45% lower risk of HCC [aHR 0.55 (0.46–0.67)]. This finding may result from the ability of AGIs to attenuate insulin resistance and decrease blood insulin levels, which may diminish HCC risk (Rudovich et al., 2011; Singh et al., 2018).

Although diabetes increases mortality in patients with compensated liver cirrhosis (Ahmadieh and Azar, 2014; Garcia-Compean et al., 2009; Elkrief et al., 2016), most patients with coexisting diabetes and cirrhosis die of hepatic failure, not diabetic complications (Petrides et al., 1994). The present study revealed that AGI use was associated with a lower risk of all-cause mortality [aHR 0.63 (0.56–0.71)], which may be due to reduced risks of HCC, decompensated cirrhosis, and hepatic failure with AGI use.

The present study has some limitations. First, the NHIRD does not contain complete information to include family history, patient body weight, physical activity, alcohol drinking, and cigarette smoking, that may influence the investigated outcomes. Second, NHIRD does not contain information on blood glucose, hemoglobin A1C, liver function and renal function test results; therefore, we could not calculate the Child-Pugh Class and the model for end-stage liver disease (MELD) scores to classify the severity of liver cirrhosis and the treated status of diabetes. However, we assessed the CCI and DCSI scores and insulin and oral antidiabetic use to evaluate the severity of diabetes; we used the clinical diagnosis to divide participants into compensated and decompensated liver cirrhosis. Some participants with minimal hepatic encephalopathy or mild-to-moderate ascites might not be captured in outpatient claims, thus resulting in underestimation of decompensated cirrhosis to affect study results. We attempted to include all possible critical variables to balance the study and comparison groups and increase their comparability. Third, AGIs seem to be more effective in lowering blood sugar in patients with higher dietary carbohydrate ratios, and rice is the staple food in Taiwan. The participants in this study were mainly Chinese; hence, the results of this study may not apply to other ethnic groups. Finally, an observational study is always subject to unmeasured and unknown confounding factors. A causal association could not be ascertained, and prospective randomized trials are needed to verify results in the present study.

However, clinically significant findings are noted. First, this is a nationwide, population-based cohort study in the real-world setting with follow-up for as long as 18 years. Second, we provided a detailed analysis of esophageal varices with bleeding, ascites, hepatic encephalopathy, decompensated cirrhosis, hepatic failure, HCC, and mortality for a comprehensive evaluation of liver-related outcomes for AGI use in patients with diabetes and liver cirrhosis.

## 5 Conclusion

AGIs are mild antidiabetic drugs with no risk of hypoglycemia and without significant side effects. Results from the present study indicate that AGIs exhibited a lower risk of liver-related complications in patients with cirrhosis. Perhaps, AGIs are suitable drugs in treating diabetes in patients with liver cirrhosis. However, results from the present study demands confirmation by large-scale prospective studies.

## Data Availability

The data analyzed in this study is subject to the following licenses/restrictions: Data of this study are available from the National Health Insurance Research Database (NHIRD) published by Taiwan National Health Insurance (NHI) Administration. The data utilized in this study cannot be made available in the paper, the Supplementary Materials, or in a public repository due to the ‘‘Personal Information Protection Act’’ executed by Taiwan government starting from 2012. Requests to access these datasets should be directed to the NHIRD Office (https://dep.mohw.gov.tw/DOS/cp-2516-3591-113.html) or by email to stsung@mohw.gov.tw.

## Nomenclature

AGIs: Alpha-glucosidase inhibitors
T2DM: Type 2 diabetes mellitus
HCC: Hepatocellular carcinoma
HBV: Hepatitis B virus
HCV: Hepatitis C virus
