# Supplementary material for: Liver-related long-term outcomes of alpha-glucosidase inhibitors in patients with diabetes and liver cirrhosis
Source: Front Pharmacol. 2022 Dec 21;13:1049094. doi: 10.3389/fphar.2022.1049094 (PMC9812564; doi:10.3389/fphar.2022.1049094)
Supplement: Supplementary file 1 [file DataSheet1.docx]

Supplementary Material

**Supplementary Table 1. Diseases and related ICD-9-CM, ICD-10-CM codes**.

| **Disease** | **ICD-9-CM codes** | **ICD-10-CM codes** |
| --- | --- | --- |
| Type 2 diabetes mellitus | 250.xx, except 250.1x | E11 |
| Liver cirrhosis | 571.5, 571.2, or 571.6 | K70.2, K70.30, K70.31, K74.0, K74.1, K74.2, K74.60, K74.69, K74.3, K74.4, K74.5 |
| Ascites | 789.59, 789.5 | R18.8 |
| Hepatic encephalopathy | 572.2 | K72.90, K72.91 |
| Esophageal varices with bleeding | 456.0, 456.2 | I85.01 |
| Jaundice | 782.4 | R17 |
|  |  |  |
| Type 1 diabetes mellitus | 250.1x | E10 |
| Dialysis | V56.0, V56.8, V45.1 | Z49.31, Z49.32, Z99.2 |
| Hepatic failure | 570, 572.2, 572.4, 572.8 | K72.00, K72.01, K72.10, K72.11, K72.90, K76.2, K72.90, K72.91, K76.7, K76.81 |
| Liver transplant | V42.7,996.82, procedure code 50.5 | Z94.4, D89.810, D89.811, D89.812, D89.813, T81.40, T81.41, T81.42, T81.43, T81.49, 0FY00Z0, 0FY00Z1, 0FY00Z2 |
| Hepatocellular carcinoma | 155.x | C22 |
| Smoking status | 305.1, 649.0, V15.82 | F17.200, F17.201, F17.210, F17.220, F17.221, F17.290, F17.291, Z87.891 |
| Alcohol-related disease | 291, 303, 305.0, 571.0-571.3, V11.3, V79.1 | F10, K70.40, K70.41, K70.9 |
| Hepatitis B infection | 070.2, 070.3, V02.61 | B16.2, B16.1, B16.0, B16.9, B18.0, B18.1, B19.10, B19.11, Z22.51 |
| Hepatitis C infection | 070.41, 070.44, 070.51, 070.54, 070.70, 070.71, V02.62 | B17.10, B17.11, B19.20, B19.21, B18.2, Z22.52 |
| Hypertension | 401–405 and A26 | I10, I11, I12, I13, I15, N26.2 |
| Dyslipidemia | 272 | E71.30, E71.31, E71.32, E71.39, E75.21, E75.22, E75.23, E75.24, E75.25, E75.29, E75.3, E75.4, E75.5, E75.6, E77, E78.0, E78.1, E78.2, E78.3, E78.4, E78.5, E78.6, E78.70, E78.71, E78.72, E78.79, E78.8, E78.9 |
| Chronic kidney disease | 250.4x, 403.xx, 404.xx, 585.xx, 586.xx, 581.8x, 791.0x, 593 | E10.2, E10.65, E11.2, E11.65, E13.2, I12, I13, N03, N08, E10.21, E11.21, N05, N06, N07, N14, N15.0, N15.8, N15.9, N16, N17.1, N17.2, N18, N19 |
| Chronic obstructive pulmonary disease | 491, 492, or 496 | J41, J42, J44, J43, or J44.9 |

| **Supplementary Table 2. Hazard ratios (HRs), and 95% confidence intervals (CIs) for outcome between AGI users and nonusers.** | | | | | | | | | |
| --- | --- | --- | --- | --- | --- | --- | --- | --- | --- |
| **Outcomes/Variables** | **n** | **PY** | **IR** | **cHR** | **95% CI** | **p-value** | **aHR^a^** | **95% CI** | **p-value** |
| **Death** |  |  |  |  |  |  |  |  |  |
| AGI nonusers | 684 | 7121 | 96.06 | 1.00 | (reference) | - | 1.00 | (reference) | - |
| Acarbose | 595 | 9045 | 65.79 | 0.67 | (0.6, 0.74) | <0.001 | 0.63 | (0.56, 0.71) | <0.001 |
| Miglitol | 14 | 244 | 57.33 | 0.59 | (0.35, 1) | 0.0517 | 0.63 | (0.37, 1.08) | 0.0931 |
| **Hepatocellular carcinoma** |  |  |  |  |  |  |  |  |  |
| AGI nonusers | 270 | 6716 | 40.20 | 1.00 | (reference) | - | 1.00 | (reference) | - |
| Acarbose | 196 | 8645 | 22.67 | 0.56 | (0.46, 0.67) | <0.001 | 0.56 | (0.47, 0.68) | <0.001 |
| Miglitol | 4 | 242 | 16.51 | 0.39 | (0.15, 1.06) | 0.0639 | 0.38 | (0.14, 1.02) | 0.0557 |
| **Decompensated cirrhosis** |  |  |  |  |  |  |  |  |  |
| AGI nonusers | 313 | 6754 | 46.34 | 1.00 | (reference) | - | 1.00 | (reference) | - |
| Acarbose | 298 | 8441 | 35.31 | 0.77 | (0.66, 0.91) | 0.0015 | 0.75 | (0.63, 0.88) | <0.001 |
| Miglitol | 8 | 235 | 34.09 | 0.69 | (0.34, 1.39) | 0.299 | 0.72 | (0.35, 1.47) | 0.3717 |
| **Hepatic encephalopathy** |  |  |  |  |  |  |  |  |  |
| AGI nonusers | 226 | 6954 | 32.50 | 1.00 | (reference) | - | 1.00 | (reference) | - |
| Acarbose | 214 | 8716 | 24.55 | 0.76 | (0.63, 0.92) | 0.0042 | 0.73 | (0.6, 0.88) | <0.001 |
| Miglitol | 5 | 240 | 20.86 | 0.6 | (0.25, 1.46) | 0.2603 | 0.6 | (0.24, 1.47) | 0.2615 |
| **Hepatic failure** |  |  |  |  |  |  |  |  |  |
| AGI nonusers | 263 | 6907 | 38.08 | 1.00 | (reference) | - | 1.00 | (reference) | - |
| Acarbose | 254 | 8670 | 29.30 | 0.77 | (0.65, 0.92) | 0.0033 | 0.74 | (0.62, 0.88) | <0.001 |
| Miglitol | 8 | 239 | 33.48 | 0.81 | (0.4, 1.65) | 0.5677 | 0.78 | (0.38, 1.6) | 0.4991 |
| AGI, alpha-glucosidase inhibitor; PY, person-years; IR, incidence rate per 1,000 person-years; cHR, crude hazard ratio; aHR, adjusted hazard ratio. | | | | | | | | | |
| aHR**^a^** multivariable analysis including sex, age, smoking, comorbidities, medications, CCI and DCSI as shown in Table 1. | | | | | | | | | |
